# Advances in circulating microRNAs as diagnostic and prognostic markers for ovarian cancer

**DOI:** 10.7497/j.issn.2095-3941.2013.03.001

**Published:** 2013-09

**Authors:** Hong Zheng, Jia-Yu Liu, Feng-Ju Song, Ke-Xin Chen

**Affiliations:** Department of Epidemiology and Biostatistics, Tianjin Medical University Cancer Hospital and Institute, National Clinical Research Center of Cancer, Key Laboratory of Breast Cancer Prevention and Therapy, Tianjin 300060, China

**Keywords:** Ovarian neoplasms, microRNAs, biological markers, early diagnosis

## Abstract

Ovarian cancer is one of the most lethal malignant gynecological tumors. More than 70% of patients with ovarian cancer are diagnosed at advanced stage. The 5-year survival in patients with advanced ovarian cancer is less than 30% because of the lack of effective biomarkers for diagnosis, prognosis, and personalized treatment. MicroRNA (miR) is a class of small noncoding RNAs that negatively regulate gene expression primarily through post-transcriptional repression. Many studies on tissue miR in ovarian cancer have been carried out and show great potential in clinical practice. However, tissue samples are not easily available because sampling causes injury. Researchers have started to focus on plasma/serum miR, assuming that blood samples may replace tissue samples in miR research in the future. Plasma/serum miR research is still in its early stages. Studies on its function in the early diagnosis of ovarian cancer have achieved some progress, but plasma/serum miR profiling for prognosis and personalized treatment of ovarian cancer remains unknown. A thorough understanding of the function of plasma/serum miR in ovarian cancer will facilitate early diagnosis and improve treatment for ovarian cancer.

## Introduction

Ovarian cancer is one of the most lethal malignant gynecological tumors. Its incidence rate ranks the second among malignant tumors of the genital system following uterine corpus cancer, but its mortality rate was the highest^1^. Given that ovarian cancer is located deep within the pelvis and is difficult to touch, as well as the lack of typical early symptoms and effective diagnostic methods, more than 70% of patients are diagnosed at advanced stage. At this stage, the tumor has metastasized into the peritoneal cavity or to distant locations^2^. Although surgical treatment and chemotherapy of ovarian cancer have improved in recent years, the prognosis of ovarian cancer remains poor. The 5-year survival in patients with advanced ovarian cancer is less than 30%^2^. Statistics from the Tianjin Medical University Cancer Institute and Hospital showed that the incidence of ovarian cancer is 9.71/100,000. It ranks the sixth in female incidence rate of malignant tumors. Ovarian cancer ranks the eighth in female cancer mortality, with a rate of 2.59/100,000 (2004, Tianjin)^3^. Early diagnosis and treatment to prevent and manage ovarian cancer is significant.

Current diagnostic methods for ovarian cancer mainly include pelvic examination, transvaginal ultrasound, and serum CA125. However, these methods are not sensitive or sufficiently specific to diagnose ovarian cancer at an early stage. Consequently, finding a diagnostic marker with high sensitivity (SN) and specificity (SP) for early detection remains a major clinical challenge.

MicroRNA (miR) is a class of evolutionarily conserved 22-nucleotide noncoding RNAs. These small molecules bind to the 3’untranslated regions of their target mRNAs, mediating translational repression and/or mRNA degradation. Thus, they function as negative regulators of gene expression^4^. MiR was first discovered in Caenorhabditis elegans. Thousands of miRs are involved in multiple biological processes, including cell differentiation, proliferation, apoptosis and metabolism. Certain miRs are involved in the pathogenesis of tumors and function as oncogenes (oncomiRs) or tumor suppressors^5^. A comprehensive and systematic understanding of ovarian cancer-specific miR profiling for early diagnosis, prognosis, and personalized treatment of ovarian cancer would be very beneficial.

Early studies on cancer-specific miR expression profiling were only confined to tumor tissue samples. A number of miRs aberrantly expressed in ovarian cancer tissues have important functions in cancer occurrence and development. Thus, miRs may represent a new approach for the detection, diagnosis, and therapy of this deadly disease. However, tissue samples are not easily accessible, which hinders the application of miR in ovarian cancer diagnosis and prognosis. Latest research shows that miR can pass between cells or tissues and organs through blood circulation. Circulatory miRs are mainly from monocytes, plasma, and exosomes^6^^,^^7^; they are resistant to degradation of RNase enzyme and are stable in the blood^8^. These characteristics make miRs potentially valuable as novel biomarkers for the detection of early cancer.

Given that blood samples can be easily obtained and have the advantages of minimally invasive continuous *in vitro* testing and high reproducibility, determining disease-specific miRs in peripheral blood to predict and diagnose cancer has become the focus of many studies. This article reviews the development of circulating miRs in ovarian cancer.

## Advances in plasma/serum miRs and discovery of plasma/serum miRs

Chim *et al.*^9^ investigated circulating fetal nucleic acids in maternal plasma in 2007. They discovered that four abundant placental miRs (miR-141, miR-149, miR-299-5p, and miR-135b) could be detected in maternal plasma during pregnancy, and showed a reduction in post-delivery plasma. Plasma miR-141 increases in the third-trimester of pregnancy. To some degree, this finding may indicate an increase of the placenta size or the miR-141 level in the placenta. Their findings may indicate a new class of biological markers for pregnancy monitoring. Lawrie *et al.*^10^ also found that miR-155, miR-210, and miR-21 are higher in serum from diffused large B-cell lymphoma (DLBCL) patients compared with normal controls, and high miR-21 expression is associated with relapse-free survival. This first description of serum miR in cancer patients suggests that miR has the potential as a non-invasive diagnostic marker for DLBCL and possibly for other cancers. Mitchell *et al.*^6^ isolated 18 to 24 nt RNA fractions from the human plasma sample of a healthy donor. Compared with real-time polymerase chain reaction amplification-generated small RNA cDNA library, they found that 27 of the 125 clones sequenced from this library corresponded to spiked-in size marker oligos or linker-linker dimers. Ninety-one of the other 98 sequences (93%) corresponded to known miRs provide direct confirmation that mature miR is presented in human plasma. Further studies showed that endogenous plasma miR exists in a remarkably stable form and is resistant to plasma RNase activity. After incubation at room temperature for 24 h or eight freeze-thaw cycles, plasma miR did not significantly change, and measurements obtained from serum and plasma were strongly correlated. They established a mouse prostate cancer xenograft model system. Plasma miR-629 and miR-660 were difficult to detect in the control mice (no known mouse homologs), whereas they were easily detected in all of the xenograft mice. The levels of these miRs were moderately correlated with tumor mass, indicating that the plasma miR may be derived from the tumor. Subsequent research in miR-141 showed the greatest differential expression in prostate cancer plasma compared with normal control, and was moderately correlated with the prostate specific antigen levels. The results extend the concept that peripheral circulating miR can serve as a marker for human cancer detection. Compared with former studies, Mitchell’s team comprehensively explained the SP and stability of plasma miR. Researchers have gradually detected plasma/serum-specific miR expression profiling in liver, lung, colorectal, ovarian, and other cancers.

## Detection of plasma/serum miRs

A variety of effective miR detection methods exist. We selected the most appropriate method based on the research purpose and sample type. Cloning and sequencing remain the primary choices in discovering new miR^11^^,^^12^. Northern blot is an important tool to verify and validate miR, but the method does not apply to high-throughput detection of clinical samples because it is complicated and has low SN^13^. Microarray chip technology is quick and has high-throughput^14^. A variety of miR chips are available, but their reproducibility and accuracy are relatively low. Thus, they are generally used for screening, and their results need to be validated by RT-quantitative PCR  (RT-qPCR)^15^. RT-qPCR analysis can accurately quantify the plasma/serum miR expression. Various PCR-based miR detection methods exist, such as stem-loop RT-PCR and polyA tailed RT-PCR^16^. RT-PCR is the most common and effective method in quantitative detection of plasma/serum miR. Driskell *et al.*^17^ established a surface-enhanced Raman scattering platform to detect and classify miR. Kato *et al.*^18^ developed a novel fluorescent DNA probe to detect mature miRs with high SP. The miR detection limit has currently reached fmol level. With further research, miR detection methods will continue to be improved and standardized. A circulating miR detection method with high SN and accuracy will eventually be established.

In summary, a large number of studies have found that plasma/serum miR has an important function in tumorigenesis, development, invasion, and metastasis, and has potential as a new class of diagnostic markers and therapeutic targets. A study on the relationship between plasma/serum miR and ovarian cancer will serve as an important guide for early diagnosis, clinical prognosis, and personalized ovarian cancer treatment.

## Plasma/serum miRs and ovarian cancer

A number of studies have investigated plasma/serum miR expression in ovarian cancer. To clearly present these important data, we summarized the differentially expressed miRs of each study in **Table 1**.

**Table 1:** Results of circulating miRs in ovarian cancer diagnosis

Author	Year	Sample types	Upregulated	Downregulated	Tumor type	Control
Douglas D. Taylor	2008	Exosomal (Serum) miRNA	MiR-21, miR-141, miR-200a, MiR-200c, miR-200b, MiR-203, miR-205 miR-214		^a^OPSC	Benign ovarian adenoma
Kimberly E. Resnick	2008	Serum miRNA	MiR-21, miR-92, miR-93, MiR-126 miR-29a	MiR-155, miR-127 MiR-99b	^b^EOC	Healthy controls
SFM Hӓusler	2010	Blood-derived miRNA	MiR-30c1	MiR-342-3p, MiR-181a, MiR-450b-5p	87.5% relapsed ^c^SOC	Healthy controls
Casina WS Kan	2012	Serum miRNA	MiR-200a, miR-200b, MiR- 200c		^c^SOC (High-grade)	Healthy controls
Ye-Won Chung	2013	Serum miRNA		MiR-132, miR-26a, Let-7b, miR-145	^c^SOC	Healthy controls
Swati Suryawanshi	2013	Plasma miRNA	MiR-16, miR-21, miR-191		^d^EAOC	Healthy controls
			MiR-16, miR-191, miR-4284		^c^SOC	

^a^OPSC, ovarian papillary serous cystadenocarcinoma; ^b^EOC, epithelial ovarian cancer; ^c^SOC, serous ovarian cancer; ^d^EAOC, endometriosis-associated ovarian cancer.

## Plasma/serum miRs and early diagnosis of ovarian cancer

Given the stability of miRs in the peripheral blood and tumor-specific miR profiling, plasma/serum miR has the potential as a non-invasive screening marker for early ovarian cancer.

Exosomes are small (50-100 nm) membrane vesicles of endocytic origin that have an important function in intercellular communication. They are released by both tumor and normal cells and can be found in various body fluids. Tumor-derived exosomes carry functional proteins, mRNAs, and miRs, and thus, could serve as novel platforms for tumor diagnosis and prognosis^19^. In 1979, Taylor *et al.*^8^ initially laid the foundation of tumor-derived exosome research in peripheral blood circulation of women diagnosed with ovarian cancer. Since then, other cells have been confirmed to release exosomes, including dendritic cells, T cells, B cells, epithelial cells, reticulocytes and embryonic cells^20^^,^^21^. Their presence in peripheral blood circulation appears to be confined to pregnancy and cancer^22^^,^^23^. The origin of circulating exosomes in cancer patients is the tumor, expressing antigens that indicate the originating tumor cells and the miR signature of their parental tumor. In 2008, they investigated exosomes from the peripheral blood circulation of ovarian cancer patients. They found that epithelial cell adhesion molecule (EpCAM)-positive exosomes are significantly distinct from profiles of benign disease or normal controls, and increase as the stage progresses. Based on former miR research on ovarian cancer tissues, they investigated the correlation of those results with exosomal-derived miRs. They found that the eight overexpressed miRs in human ovarian cancer tissue^24^ (miR-21, miR-141, miR-200a, miR-200c, miR-200b, miR-203, miR-205, and miR-214) are also elevated in blood-derived exosomes. Comparisons between tumor-derived miR profiles and peripheral circulation-derived exosomal miRs indicate that they are not statistically different. They failed to demonstrate the eight differentially expressed exosomal miRs in terms of tumor stage or grade. Some other miRs appeared to be different between early and late stages of ovarian cancer. A larger-scale study that included additional confounding factors is needed to define their significance. However, in all cases, these miR levels are significantly elevated compared with exosomes derived from benign disease. This result indicates that blood-derived exosomal miR may serve as a novel platform for tumor diagnosis and prognosis.

Exosomes are usually collected by anti-EpCAM-coupled magnetic beads, but Rupp *et al.*^19^ found that CD24 is present and EpCAM is absent from serum exosomes in breast cancer patients. This finding may suggest that EpCAM can be cleaved from exosomes by serum metalloproteinase. A verification of whether or not Taylor *et al.* missed some exosomes is not possible. Loss of EpCAM on serum exosomes may hinder enrichment by immune-affinity isolation. This phenomenon may suggest that an additional marker, such as CD24, should be added for tumor-derived exosome enrichment from blood in the future.

Resnick *et al.*^25^ empirically selected 21 miRs from the expression profile for serum miR examination by RT-PCR. Of these 21 miRs, 10 are common to published ovarian cancer profiles. Upon follow-up RT-qPCR of the 21 miRs, five are overexpressed (miR-21, miR-29a, miR-92, miR-93, and miR-126) and three are underexpressed (miR-127, miR-155, and miR-99b) in the serum of ovarian cancer patients compared with normal controls. These three miRs, namely, miR-21, miR-92 and miR-93, with the highest serum expression are significantly overexpressed in three patients with normal pre-operative CA-125 level. Among the five overexpressed miRs that they discovered, three are potential oncomirs (miR-21, miR-92, and miR-93). Contrary to published ovarian cancer profiles, they significantly demonstrate overexpressed miR-29a and miR-126  in the serum from ovarian cancer patients. A number of tumor suppressor activity reports on miR-126 and miR-29a exist. Overexpression of these miRs in ovarian cancer tends to suggest that they behave as oncomirs, and serum miRs are disease specific. Their research is the first to describe the use of RT-PCR microarray platform to obtain a miR profile on serum RNA. Given the limited small-sample size and the lack of long-term outcome data, whether these serum miRs are necessarily tumor-derived remains unclear. They presented a pilot study demonstrating the potential utility of serum miR. The correlation of miR status with progression-free interval or survival needs to be studied further. Chung *et al.*^26^ analyzed total RNA isolated from the serum, tissue, and ascites of serous ovarian cancer by a microarray. They sorted out several miRs showing a consistent regulation tendency throughout all three specimens and the greatest range of alteration in serum as potential biomarkers. Five miRs (miR-132, miR-26a, let-7b, miR-145, and miR-143) were identified to be the most markedly downregulated miRs in serum from ovarian cancer patients compared with those of the controls. Four miRs (miR-132, miR-26a, let-7b, and miR-145) were significantly underexpressed in the serum of ovarian cancer patients detected by quantitative RT-PCR, which could be considered as potential novel biomarkers for serous ovarian cancer.

Compared with previous research on miR in serum or plasma, Hӓusler *et al.*^7^ investigated whole blood-derived (including cellular fraction) miR of ovarian cancer. Blood-derived miR can be found as free circulating nucleic acids or mononuclear cells. Of the 147 significantly deregulated miRs, miR-30c-1, miR-191, miR-155, miR-16, miR-106b, miR-146a, miR-29a, and miR-383 are related to ovarian cancer, perfectly in accordance with previously described alterations in ovarian cancer tissue research. By contrast, the other 15 miRs are not linked to a specific disease. They believed that regulatory T cells^27^ or stromal and myeloid progenitors^28^^,^^29^, which were recruited to the tumor site, may significantly contribute to these profiles. Considering that the formation of a pre-metastatic niche by hematopoietic cells is an early event in tumorigenesis and metastasis^30^, detectable ‘imprinted’ profiles of blood cells at the very beginning of tumor development seem plausible. The miR released from cancer cells is only detectable until a significant neoplastic mass has accumulated. This finding explained the differences between blood miR and miR from tissues and the significant differences in miR profiling of various malignancies. The results strongly suggest that the observed patterns are disease specific instead of nonspecific systemic inflammatory reaction or therapy-related toxicity. Therefore, further development is promising. The limitation of this study is its restricted size, which made the results sensitive to individual outliers. A larger study cohort that considers age, menopausal status, chemotherapy, and histopathological and molecular characteristics is warranted, which may significantly increase accuracy, SN, and SP.

## Plasma/serum miRs and classification of ovarian cancer

At the beginning of plasma/serum miR study, researchers always try to verify whether plasma/serum miR and miR in the tissue are in good agreement so plasma/serum miR profiling could be used as a surrogate for tissue miRs. The usual approach is screening plasma/serum miR based on tissue-specific ovarian cancer miR profiling instead on genome-wide miR detection. Suryawanshi *et al.*^31^ reported several novel findings on plasma miR in endometriosis-associated ovarian cancer (EAOC) based on global profiling: (1) distinct miR expression profiles between tissue and plasma observed may suggest that disease tissue or malignant tumor cells are not the sole source of plasma miR. Important information will be missed without independent global miR profiling of circulating miRs; (2) epithelial ovarian cancer (EOC) is a highly heterogeneous disease with regard to histopathological and molecular characteristics that consist of four major histotypes, namely, serous, mucinous, clear cell and endometrioid^32^. The four main histologic subtypes of ovarian cancer are now considered different diseases, which may develop and respond to chemotherapy differently, and are characterized by distinct mRNA expression profiles. In this case, these subtypes should be treated as four different diseases instead of one single entity, which may be beneficial in finding more specific diagnostic indicators. This concept is further supported by their results, EAOCs and SOCs are different clinical entities that can be distinguished based on plasma miR expression profiles; (3) a general trend of elevated plasma miR expression from healthy controls to endometriosis to EAOCs exists, but not in SOC samples, suggesting that these miRs may serve as novel biomarkers that indicate pathological progression from benign to precursor lesion to fully developed EAOCs. By analyzing different combinations of 23 candidate miRs, three plasma miRs, namely, miR-16, miR-191 and miR-195, were all found to be upregulated in endometriosis, thus enabling differentiation between healthy and endometriosis samples (88% SN, 60% SP). A combination of miR-16, miR-21, and miR-191 can differentiate between healthy control and EAOCs (86% SN, 85% SP), whereas miR-21, miR-362-5p, and miR-1274a can differentiate between endometriosis and EAOCs (57% SN, 91% SP). MiR-21, miR-191, and miR-1975 together could distinguish between EAOC and SOC (86% SN, 79% SP). The expression signature of miR-16, miR-191, and miR-4284 could be used to discern healthy individuals from patients with SOCs (90% SN, 55% SP), whereas miR-362-5p, miR-628-3p, and miR-1915 can differentiate between endometriosis and SOCs (90% SN, 73% SP). The reported SN and SP values of the plasma miR signatures are lower than the required SN of at least 75% and SP of more than 99.6% for clinical applications. Given that few published studies on specific plasma miRs of various subtypes of ovarian cancer are available, their potential for ovarian cancer classification needs to be investigated further. The plasma miR signatures they identified, which were observed in an EOC mouse model, further certified the possible usage of plasma miR as a promising biomarker of ovarian cancer detection or even classification. Chung *et al.*^26^ sorted several miRs that showed consistent regular tendency in all three specimens (serum, tissue, and ascites) and established a specific miR signature limited to the serous subtype of ovarian cancer. Their serum miR profiles were distinct from previously reported studies^7^^,^^8^, but a larger scale study, involving other subtypes, is needed to verify their significance.

## Plasma/serum miRs and prognosis and personalized treatment of ovarian cancer

Plasma/serum miR research is still in its early stage, and few references can be found in prognosis and personalized treatment of ovarian cancer.

Many studies on miR in ovarian cancer tissue have been carried out, and are mainly related to miR-200 and let-7 families^33^. The function of the miR-200 family in ovarian carcinoma is not clearly elucidated. On one hand, miR-200 family members are believed to be metastasis suppressors, and most of the studies performed on the family are about their overexpression in ovarian cancer. On the other hand, some studies demonstrated that miR-200 family members are downregulated^34^ or even unchanged^35^. These diverse results may be due to the inclusion of ovarian stromal cells lacking miR-200 expression or differences in normal controls^36^. Most of the let-7 family members are confirmed to be tumor suppressors^37^. The detected abnormal expression of plasma/serum miR is mainly from the miR-200 family, including miR-200a, miR-200b, miR-200c, miR-141 and miR-429.

Kan *et al.*^38^ extracted highly expressed miR-200a, miR-200b, and miR-200c in the plasma of serous ovarian cancer. Taylor *et al.*^8^ found that miR-141, miR-200a, miR-200c, and miR-200b are overexpressed in blood-derived exosomes. However, these researches^8^^,^^38^ lacked early-stage disease plasma/serum samples and long-term follow-up data. Thus, no consensus has been reached on the relationship of peripheral tumor-associated miR with metastasis, recurrence, and survival of ovarian cancer. We believe that the application of plasma/serum miR-200 family in clinical prognosis, therapeutic effect, and tumor recurrence monitoring of ovarian cancer patients will be further developed.

The standard treatment for advanced ovarian cancer is surgical tumor debulking, followed by platinum-based chemotherapy^39^. To date, few effective treatments for advanced ovarian cancer patients are available mainly because of the molecular heterogeneity of ovarian tumor tissue, which has also led to different clinical effects^32^. Studies are needed to find predictive and prognostic markers to help optimize and personalize treatment of ovarian cancer and to improve the therapeutic effect.

MiR functions as an oncogene or tumor suppressor. For miR with oncogenic character, anti-miR oligonucleotides, “miR-sponges”, or “miR making” can be used to silence their oncogenic activity^40^. The expression of tumor suppressor miR can be restored with engineered viral vector approach for anti-cancer therapy^41^. Considering the risk of insertional mutagenesis and the toxicity of viral vector application, the application of miR mimics is a promising alternative for the therapeutic restoration of candidate miR in cancer cells. MiR mimics constitute double-stranded and chemically modified miR molecules, which can be transiently transfected into target cells in precursor form, where they can resume functioning as tumor suppressors^42^.

Studies show that a great potential of tumor tissue miR exists in drug resistance and anti-cancer therapy prediction^43^^-^^45^. Given that miR are also present in the blood, we propose that plasma/serum miR expression profiling can provide personalized treatment information, such as monitoring of treatment effects and predicting drug resistance, in addition to diagnosis and prognosis of ovarian cancer. Plasma/serum miR seems more reliable and more sensitive compared with traditional markers, such as mRNA and protein, because conventional markers are often disproportionate and easily degraded in the blood. However, the relationship between plasma/serum miR and individualized treatment of ovarian cancer is not clearly elucidated. Given that not all patients can tolerate surgery-derived gene analysis, plasma/serum miR has the potential for molecular prediction of therapeutic effects as a non-invasive marker. It also helps in determining treatment processes and therapeutic targets of ovarian cancer patients in this new field. This new method will be used to identify different subtypes of ovarian cancer patients who may be sensitive or resistant to certain drugs. More importantly, we will be able to effectively prevent the occurrence of secondary drug resistance by detecting changes in plasma/serum miR expression level in ovarian cancer. A large number of controlled clinical trials is needed to confirm whether plasma/serum miR is a novel marker for adjustment of dosage regimen, personalized treatment, and therapeutic targets.

## Conclusion

The cause of ovarian cancer is not fully understood to date, which could be attributed to several factors, including fertility, ovulation drugs, environmental factors and genetics. Although ultrasound, laparoscopy, cytology, and serum CA125 remain the major approaches for ovarian cancer detection, their invasiveness and low SN and SP hinder most of their clinical use. Studies show that the use of plasma/serum miR as a non-invasive marker of ovarian cancer is expected for early diagnosis and prognosis monitoring, and also has great potential in individualized treatment. Attention should be focused in the clinical use of plasma/serum miR. (1) Plasma/serum miR expression profiling varies significantly with changed physiological or pathological condition, such as pregnancy, heart failure or sepsis^9^^,^^46^^,^^47^. (2) In experimental phase, the control group generally consists of healthy people without family history of related diseases. The situation is often more complicated in population-wide clinical screening, and whether plasma/serum miR can exhibit good SN and SP still need to be investigated. (3) The screened out tumor-associated plasma/serum miRs lack further experimental demonstration, and the reference range is not determined. (4) Ruptured erythrocytes release inhibitors of RT-PCR reaction. To control other factors, selecting the most sensitive economical detection methods and the most specific miR combination is the major challenge in clinical practice. Further studies will gradually solve these problems. Plasma/serum miR will become a novel molecular detection and treatment biomarker because of its broad application prospects in future clinical diagnosis and prognosis of ovarian cancer.
